# Takayasu's Arteritis with Systemic Lupus Erythematosus: A Rare Association

**DOI:** 10.1155/2015/934196

**Published:** 2015-06-15

**Authors:** Dhrubajyoti Bandyopadhyay, Vijayan Ganesan, Debarati Bhar, Diptak Bhowmick, Sibnarayan Sasmal, Cankatika Choudhury, Sabyasachi Mukhopadhyay, Adrija Hajra, Manas Layek, Partha Sarathi Karmakar

**Affiliations:** ^1^Department of Accident and Emergency, Lady Hardinge Medical College, New Delhi, India; ^2^Department of Internal Medicine, KB Hostel, RG Kar Medical College, Room No. 38, Khudiram Bose Sarani, Kolkata, West Bengal 700004, India; ^3^Department of Internal Medicine, RG Kar Medical College, Kolkata, India; ^4^Department of Internal Medicine, KB Hostel, RG Kar Medical College, Room No. 1, Khudiram Bose Sarani, Kolkata, West Bengal 700004, India; ^5^Department of Internal Medicine, Girls Hostel, RG Kar Medical College, Room No. 22, Khudiram Bose Sarani, Kolkata, West Bengal 700004, India; ^6^RG Kar Medical College, Kolkata, India; ^7^Department of Internal Medicine, IPGMER, Kolkata, India; ^8^Lady Hardinge Medical College, New Delhi, India

## Abstract

We report the case of a 24-year-old nondiabetic, nonhypertensive lady with history of fatigue, dyspnoea and limb claudication. She has been diagnosed with Takayasu's arteritis. Subsequently she developed rash, alopecia, joint pain, and various other laboratory abnormalities which led to a diagnosis of SLE. Takayasu's arteritis (TA) rarely coexists with systemic lupus erythematosus (SLE). The absence of specific SLE markers in patients with TA who subsequently develop SLE suggests that the coexistence of these conditions may be coincidental. The antiphospholipid syndrome in patients with SLE may mimic the occlusive vasculitis of TA.

## 1. Introduction

Takayasu's arteritis involves narrowing or obliteration of the major arteries and branches of the aorta resulting in the terminology “pulseless disease” [1]. Tuberculosis, syphilis, rheumatic fever, systemic lupus erythematosus (SLE), and other autoimmune diseases have been implicated in its etiology. So far, 20 cases of aortitis syndrome coexisting with SLE have been described, of which 4 had associated antiphospholipid antibody syndrome (aPLS) [1]. Patients with TA and/or SLE have similar age of onset and female predominance. Here we report a case where SLE was preceded by TA.

## 2. Case Report

**Figure 1:**
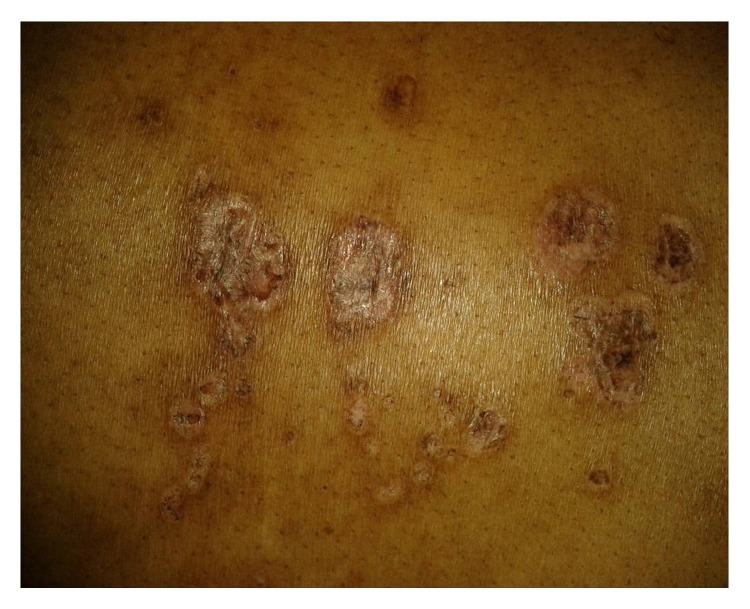
Discoid rash over the back.

A 24-year-old unmarried Indian woman consulted her doctor for dyspnoea, fatigue, and upper limb claudication 3 years ago. She was diagnosed as a case of Takayasu's arteritis depending on impalpable pulse and unrecordable blood pressure on left arm, carotid artery bruit on left side, low velocity biphasic flow of all arteries of both upper limbs in Doppler study, and high erythrocyte sedimentation rate (ESR) (60 mm/hr). Antinuclear antibody (ANA) report was negative. She was on daily oral prednisolone (10 mg/day) but she was not adherent to the treatment and follow-up. After 3 years she presented with small and large joints arthritis, malar rash, discoid rash, oral ulcer, nonscarring alopecia, and photosensitivity (Figure 1). Peripheral pulses were not palpable over left brachial and radial artery, and blood pressure was 110/70 mm Hg in the right upper limb and both the lower limbs, not recordable in the left upper limb.

**Figure 2:**
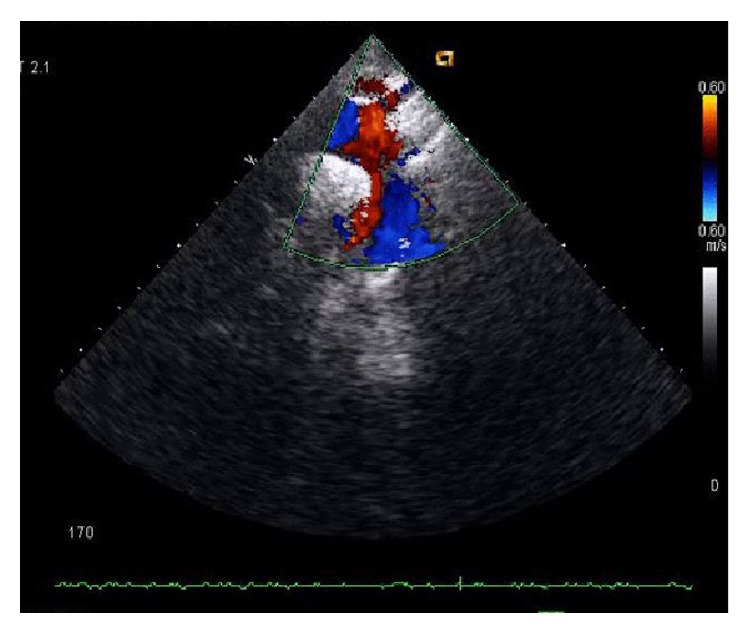
ECHO-Doppler study showing stenosis of the right subclavian artery.

**Figure 3:**
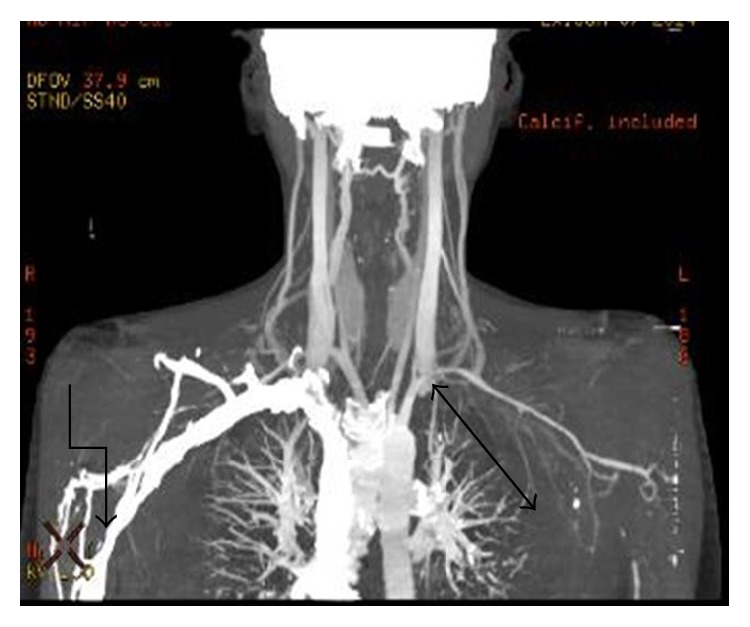
CT angiography showing near total stenosis of the proximal part of left subclavian artery and luminal obstruction of left common carotid artery and more than 50% stenosis of 3rd part of the right subclavian artery.

Laboratory investigations showed hemoglobin 8.3 gm/dL, reticulocyte count 6%, and erythrocyte sedimentation rate (ESR) 60 mm/hr. Urine for 24 hr protein was 2.41 gm/day. Serum antinuclear antibody (ANA) which was negative 3 years back became positive in high titre (1 : 320, homogeneous pattern) and serum anti-dsDNA (titre was >1 : 180) came out to be positive. Further serum analysis revealed Blood Urea Nitrogen (BUN) 67 mg/dL, creatinine (Cr) 2.3 mg/dL, CRP 6.0 mg/L, C3 and C4 decrease, and positive anti-Sm antibody. Prothrombin time and activated partial prothrombin time were normal. Direct Coombs test was positive and antiphospholipid antibody was negative. Chest X-ray and ultrasonography of abdomen were normal. Echocardiography showed chink of pericardial effusion. Echo-Doppler study revealed that (1) right subclavian artery cannot be visualized beyond proximal part with wall thickening and mild luminal narrowing of right common carotid artery. (2) There were proximal stenosis of left subclavian and left common carotid artery (Figure 2). On CT angiography there was near total stenosis of the proximal part of left subclavian artery. Luminal obstruction of left common carotid artery and more than 50% stenosis of 3rd part of the right subclavian artery were noted (Figure 3). Renal biopsy was consistent with lupus nephritis IV-G (A) according to ISN/RPS (International Society of Nephrology and Renal Pathology Society) classification.

According to SLEDAI (SLE Disease Activity Index), the patient had a score of 22, so the patient had a severe flare. Then we put the patient on steroid at a dose of 1 mg/kg body weight and intravenous monthly cyclophosphamide. Joint pain, dyspnea, and fatigue were subsided within 2 months of treatment.

Serum reports showed BUN 22 mg/dL, Cr 1.4 mg/dL, and CRP 2.0 mg/L and urine for 24 hr protein was 300 mg/day after 2 months of treatment.

## 3. Discussion

Takayasu's arteritis is more prevalent in Japan and Afro-Asian countries. Autopsy incidence in Japan is quoted as 33% [2]. Lupi-Herrera et al. found ANA in 6% of cases of Takayasu's arteritis and LE cells in 2% of cases [3]. Most of the cases reported so far in literature had aortic aneurysms or dissections detected at autopsy or during surgery for dissections [4].

All of the American College of Rheumatology criteria of Takayasu's arteritis [5] were met which included (1) age < 40 years; (2) decreased left brachial artery pulse; (3) blood pressure difference of >10 mm Hg between the two arms; (4) bruit over the subclavian artery and carotid artery; (5) limb claudication; and (6) angiographic evidence. Eleven of the Systemic Lupus International Collaborating Clinics (SLICC) [6] classifications criteria of SLE were met which included (1) malar rash; (2) oral ulcer; (3) nonscarring alopecia; (4) discoid rash; (5) photosensitivity; (6) serositis; (7) arthritis; (8) hematological disorders, anemia; (9) renal disorder, 24-hour urinary protein 2.41 g/L and biopsy proven nephritis; (10) strongly positive ANA; (11) positive anti-dsDNA (>1 : 180 titer).

Vascular disease is frequent in patients with systemic lupus erythematosus and represents the most frequent cause of death in established disease. In this context, vasculopathy can be directly etiologically implicated in the pathogenesis of the disease, presenting as an acute/subacute manifestation of lupus (e.g., antiphospholipid syndrome and lupus vasculitis). Alternatively, it can develop as an important accompanying comorbidity (steroid-related atherosclerotic disease) or represent the synergistic pathogenetic outcome of augmented atherosclerosis within a proinflammatory environment [7].

Takayasu's arteritis involves mononuclear infiltration, granulomatous change, and fibrosis in the media with intimal thickening and obliterative aortitis of the large vessels. Immune mechanisms (mainly cell-mediated immunity) probably play a major role [8]. Vasculitis may manifest in as high as 56% of lupus patients throughout their life, in contrast to antiphospholipid syndrome which has a prevalence of 15% [7]. Patients with vasculitis are mainly male and tend to be of younger age [7]. Inflammatory vascular disease is triggered by the in situ formation or the deposition of immune complexes within the vessel wall [7]. The principal manifestations of the disease were found to be associated with smaller-sized arteries. Fibrinoid degeneration, intimal thickening, thrombosis, and sclerosis were identified in SLE. However, patients with medium-sized arterial involvement usually presented with more frequent thrombotic events and exhibited higher morbidity rates than the rest of the patients [7]. Lesions of the aorta are extremely rare [9].

The claudication and asymmetric pulse preceded the appearance of rash and arthritis by more than a year. Antinuclear antibody was negative also at that time. Usually, SLE involves small and peripheral vessels but here involvement spans the medium to large vessels. There is a temporal association between Takayasu's arteritis and systemic lupus erythematosus, and aortoarteritis was the initial manifestation of systemic lupus erythematosus in our case, which is very rare. The natural history of Takayasu's arteritis is variable. But it usually progresses slowly with the development of hypertension, retinopathy, and aneurysm formation [10]. The therapeutic strategies and complications are different, and both diseases are associated with significant morbidity and mortality. Therefore, recognition of the coexistence of SLE and aortoarteritis is of prime importance.
